# Quantitative-genetic analysis of directional adaptation suggests low maximum sustainable rates of change in agreement with data from field populations

**DOI:** 10.1038/s41598-025-24445-2

**Published:** 2025-12-03

**Authors:** Mark Pagel, Jacob D. Gardner, Andrew Meade

**Affiliations:** School of Biological Sciences, University of Reading, Reading, UK

**Keywords:** Sustainable phenotypic change, Directional change, Climate change, Ecology, Environment, Ecology, Evolution, Environmental sciences

## Abstract

What rates of directional change are species likely to be capable of sustaining indefinitely such as in response to a warming climate? We derive estimates of the maximum rates of phenotypic change that populations can sustain in response to a directionally changing environment, using a quantitative genetics simulation model whose parameters are calibrated with data from natural populations. Sustainable directional change is largely limited to $\le$2–4% of a trait standard deviation per generation, in agreement with an estimate derived from quantitative-genetic theory and with published field studies. Data from thirty-seven longitudinal field-studies of species’ phenological responses to a warming climate yield rates of change that fall in the 68th–86th percentiles of our predictions for what populations can sustain, and there are suggestions that the rate of climate change may already have diminished their capacities to maintain these rates. Given the pace of climate change, species with generation times greater than four years may be especially at risk.

**Supplementary Information:**

The online version contains supplementary material available at 10.1038/s41598-025-24445-2.

## Introduction

Global climate change is placing demands on species to adapt to warming temperatures potentially for periods lasting well beyond this century^1^. This raises the question of for how long and at what rates can species be expected to sustain directional change? The question is made even more pressing because merely observing a species changing in concert with a changing climate is not sufficient to conclude that it is tracking a moving environmental optimum: environmental optima are seldom known and even though a population may change at a steady pace and thereby give the impression of successfully tracking the environment, it can be lagging further and further behind the optimum such that extinction is likely and may occur abruptly^2^.

Studies of species adapting in the wild, or in selection experiments, often reveal plentiful genetic variation in fitness^3^ and that species have the capacity to change rapidly^3–9^. But with few exceptions^10^ these studies typically observe species over one or a small number of generations. Long-term artificial selection experiments show that large and sustained responses to directional selection are possible. Laboratory populations of *Drosophila* increased abdominal bristle numbers by 13–19 standard deviations over 75–90 generations, or 0.18 to 0.22 standard deviations (*sds*) per generation^11^, and increased wind-tunnel flight speeds linearly for over 75–100 generations^12^. Maize (*Zea mays*) oil yields and other traits increased by between 8 and 27 *sds* over 70 generations^13^. On the other hand, these studies artificially return population sizes to their starting points following selection regimes that typically impose 80–95% mortality and so leave open the question of what rates can be sustained in the wild.

Genetic variance likely persists in the maize and fruit-fly populations owing to large numbers of segregating loci undergoing recombination^14–17^: mutations and new allele-combinations continually introduce variance even as existing alleles are driven to fixation by strong selection. But lacking detailed knowledge of the genetic architecture of most species’ traits^17^, the question of for how long and at what rates species can be expected to sustain directional phenotypic change can be addressed using quantitative-genetic theory and simulations. Bürger and Lynch^2^ developed a “quasi-deterministic” theoretical framework suitable for quantitative traits influenced by large numbers of loci, and used it to explore long-term adaptation in a changing environment. The model’s theoretical predictions provided good approximations to the behaviour of a simulated population subjected to varying rates of environmental change. The authors concluded that the typical maximum sustainable rate of adaptation was likely to be on the order of 10% of a phenotypic standard deviation per generation but speculated that it could be as low as 1%.

Here we generalise Bürger and Lynch’s work to provide estimates of how real-world populations might respond genetically to a directionally changing environment. We employ a multi-locus Monte Carlo quantitative-genetics simulation model whose parameters are calibrated with probability distributions derived from meta-analyses of natural populations. The goal is to reproduce a set of conditions that characterise species in the wild, asking what rates of environmental change they can accommodate and for how long, and we compare our results to predictions from theory and to real-world populations that have been undergoing long-term directional change.

We find that species with characteristics typical of those seen in the wild are largely limited in their ability to sustain genetic adaptation to ≤2–4% of a trait *sd* or less per generation and that this agrees with theory, published studies, and longitudinal field data. The 2–4% figure provides a ‘yardstick’ for assessing populations’ vulnerability to a changing climate – observed rates of change that exceed 2–4% per generation may be cause for concern, with species whose generation times are greater than around four years especially imperilled. Our results also help to calibrate methods for understanding long-term macroevolutionary change, specifically whether macroevolutionary rates of morphological change can be explained by ordinary Darwinian microevolutionary mechanisms operating within populations^18,19^ or require special burst or jump processes (e.g.,^20^).

## Results

### Characterisation of simulated populations

The quantitative trait is assumed to arise from *k* = 100, 500, 1000 or 5000 diploid loci that undergo recombination (Methods). This assignment reflects the recognition from very-large-sample-size genome-wide association studies (GWAS) that thousands of loci may affect quantitative traits such as body size ($\sim$12,000 for human height^21^) and life histories and explain the missing heritability problem (that known loci often explain only a small proportion of variability in inheritance)^21^. Systems reported in plants and animals to have a smaller number of loci of large effect tend to explain relatively small proportions of the variance in quantitative traits and are often based on smaller sample sizes that may miss loci of weak effect^22^.

Empirical work shows that when the multiple loci affecting a trait are ordered by their effects on the phenotype, their effect sizes can often be reasonably described by an exponential distribution^23^, corroborating a prediction from Orr^24^ that can be traced back to Fisher^25^. This means our use of *k* loci of equivalent effect may mimic the behaviour of a larger number. For each of the 4 loci × 2 recombination-rate combinations, we generated 15,000 populations of size *n* = 1000 with randomly varying characteristics as described below, yielding 120,000 simulated populations. A population size of *n* = 1000 is in line with effective population sizes reported in natural populations: Waples et al.^26^ report a mean effective population size of 464 ± 133 for *n* = 63 species of plants and animals, although population sizes in nature can vary over orders of magnitude (also see Discussion).

Populations were assigned a fixed set of starting conditions by randomly drawing values from five probability distributions that define a population’s characteristics and selection regimes (Table 1, Methods): the within-population variance of the phenotypic trait in natural populations $({\sigma}_{T}^{2})$, heritability of the trait (*h*^*2*^), the strength of within-population selection ($\omega,$ smaller values indicating stronger selection around the optimum), the amount by which the environmental optimum changed every generation during the period of directional selection (${{\Delta_{env}}}$), and finally the amount of new variance in the phenotypic trait arising each generation owing to genetic mutation. The mutational variance is conventionally described as the ratio of the mutational variance to the environmental variance in the trait $({\sigma}_{m}^{2}/{\sigma}_{e}^{2}),$ where values in the range of 10^−4^ to 10^−2 ^are commonly observed (e.g.,^18,27^, Methods). The probability distributions were derived from meta-analyses^6,17,27,28^ of natural and wild populations (Table 1).

This procedure means the 120,000 simulated populations numerically integrate over a space of phenotypes and selection regimes (${\sigma}_{T}^{2}$ $\times$ *h*^*2*^ $\times\:\omega\:\times$ ${\sigma}_{m}^{2}/{\sigma}_{e}^{2}\times{{\Delta_{env}}})$ representative of those observed in natural populations while respecting their relative probabilities.

**Table 1:** Parameter values in the simulation model.

Simulation parameter	Probability density	Parameters, range		References
Phenotypic variance	Exponential	0.018, range 0.01–0.09		^6,17,28^
Heritability	Weibull	1.6, 0.4, range 0.01 to 0.99		^17^
Mutational heritability,$\:{\sigma}_{m}^{2}/{\sigma}_{e}^{2}$	Gamma	1.1, 0.004		^27,29–31^
Width of fitness function,$\:\omega$	Uniform	0–10		Values chosen to yield strengths of directional selection comparable to those previously reported^6,28^
Rate of environmental change	Uniform	0–0.3		

The simulations keep track of population phenotypic and genotypic means and variances during an initial period of stabilising selection (10,000 generations) followed by directional selection until the population goes extinct or survives 240,000 generations. The period of stabilising selection ensures populations are in mutation-selection equilibrium. Information on the realised strength of directional selection, population size, and the number of generations a population survives is recorded throughout.

The numbers of loci in simulated genomes and the rate of recombination made very little quantitative difference to the results (Supplementary Tables S1–S3) and so unless otherwise specified we report all outcomes averaging over both factors. Preliminary simulations using between 1 and 50 loci returned results that converged on those with 100–5000 by around 50 loci (Table S4). The median number of generations to extinction and rate of phenotypic change for the simulations with 100–5000 loci were also captured in the interquartile ranges of results from the simulations with 1–50 loci. Sixteen simulations with very small $\omega$ (very strong selection around the optimum) went extinct prior to the onset of directional selection, leaving 119,984 populations for analysis.

We tested the simulation model against predictions^2,32^ regarding the size of genetic variances at their mutation-selection equilibrium during the period of stabilising selection and variations in population survival time as a function of the size of $\omega$ and found them in agreement with theory (Supplementary Text and Figure S1). We also assessed the intensity of our selection regimes and found they yielded strengths of directional selection comparable to those observed in field studies of natural populations (Supplementary Figure S1). Analytical and simulation modelling^2,33^ predict that the initial response to the onset of directional selection is a rapid increase in the phenotypic mean and an increase in the genetic variance over its equilibrium value, effects we also observe (Supplementary Figure S2).

### Response to a directionally changing environmental optimum

**Fig. 1:**
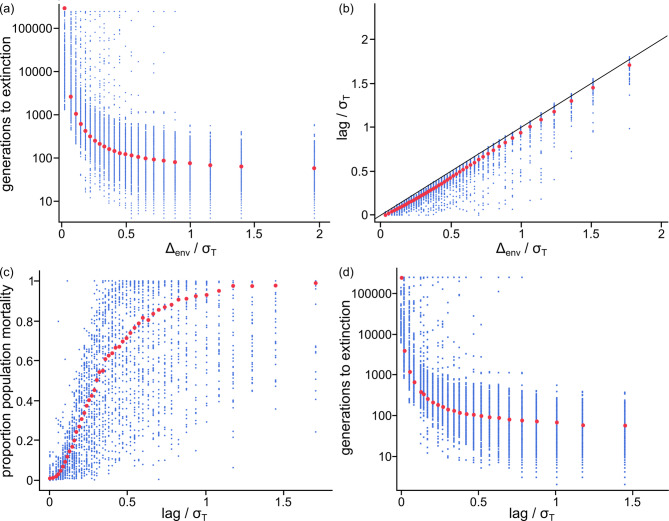
Responses to environmental change. (**a**) Median (red dots) and range (blue) of generations to population extinction versus ${{\Delta_{env}}}$ per generation expressed in units of standard deviations of the phenotypic trait (‘trait-scaled’) and divided into bins with equal numbers of populations per bin. Note x-axis: ${{\Delta_{env}}}/{\sigma_{T}}\sim7$ bin not shown; over 98% of the environmental rates fall below 2.0 $sd$*s* per generation; (**b**) Median standardised lag (red dots) and range (blue dots) measured as the trait-scaled difference per generation between the environmental optimum and the phenotypic mean during the period of directional selection divided into bins with equal numbers of populations per bin: phenotypic change lags behind ${{\Delta_{\text{e}\text{n}\text{v}}}}/{\sigma_{T}}$ (the solid 1:1 line) except for very low rates of environmental change where lag$\:\sim0$; (**c**) Median (red) and range (blue) of within-population mortality measured at generation 47 (the median number of generations to reach 50% population mortality) versus standardised lag; (**d**) Median (red) and range (blue) of the populations’ expected survival times versus standardised lag. Generations to extinction plummets at faster than a linear pace with increasing lag (best fitting log-log curve – not shown): log(*time to extinction*) = 4.17 $-$ 0.70$\:\times\:$log(*standardised lag*); best fitting quadratic curve:  log(*time to extinction*) = 4.0 $-$ 0.91$\:\times\:$log(*standardised lag*) $-$ 0.018$\:\times\:$log(standardised lag)^2^, *R*^*2 *^= 0.83; the significant quadratic term (*p *< 0.0001, two-tailed) indicates that the rate of decline accelerates with increasing lag.

How well can populations respond to a directionally changing environment? The median standardised rate of phenotypic change across all populations and selection regimes was ${{\Delta_{trait/{\sigma_{T}}}}}$ = 0.056 trait *sds* per generation (Interquartile Range [IQR] = 0.030–0.103, 97.5th percentile = 0.37, Table 2; we use medians because distributions are skewed), or equivalently 5.6% of a trait *sd* per generation (Table 2). The upper range of phenotypic rates of change we observe includes the unusually high rates reported in some artificial-selection studies^11–13^. On the other hand, and consistent with previous results^2^, the number of generations a population survives directional selection falls away rapidly as the pace of environmental change increases (Fig. 1a). The median time to extinction = 141 generations (IQR = 79–326), and long-term survival is common only for populations experiencing slow rates of environmental change.

**Table 2:** Generations to extinction and rates of environmental and phenotypic change.

Sample	Generations to extinction, median (IQR)	${\varvec{\Delta}}_{\varvec{t}\varvec{r}\varvec{a}\varvec{i}\varvec{t}}$ *sds* per generation, median (IQR)	Generations to 50% population mortality, median (IQR)	${\varvec{\Delta}}_{\varvec{t}\varvec{r}\varvec{a}\varvec{i}\varvec{t}}$ *sds* per generation 50% mortality, median (IQR)	${\varvec{k}}_{\varvec{c}}$ *sds* per generation, median (IQR)
All populations *N* = 119,984 (median ${\sigma}_{m}^{2}/{\sigma}_{e}^{2}$ =0.0038)	141 (79–326)	0.056 (0.030–0.103, 97.5th = 0.37)	47 (25–113)	0.033 (0.017–0.058)	NA
Survivors *n* = 6158 (median ${\sigma}_{m}^{2}/{\sigma}_{e}^{2}$ =0.0078) **Adjusted for** ${\mathbf{\sigma}}_{\textbf{m}}^{2}/{\mathbf{\sigma}}_{\textbf{e}}^{2}$ *	240,000	0.030735 (0.011–0.066) 0.019* **(0.007–0.052)**	98,905 (38,443 − 167,242)	0.030 (0.011–0.061)	0.022 (0.008–0.056) ${\varvec{\:\le}}{\varvec{\:50\%}}$ **mortality** **0.021** **(0.008–0.053)**

* adjusted to give survivors the same average mutational heritability as the full sample (see text).

The number of generations a population survives falls rapidly with increasing rate of environmental change (Fig. 1a) because the population phenotypic means increasingly fail to keep up with the environmental optimum (Fig. 1b): phenotypic lag, measured as the average number of trait *sds* the population’s phenotypic mean is behind the environmental optimum per generation until extinction, is close to zero – indicating that populations are keeping up with the environmental optimum – only for very slow rates of environmental change (Fig. 1b), but then rises almost linearly with ${{\Delta_{env/{\sigma_{T}}}}}$ (Fig. 1b). That is, for anything other than the slowest rates of environmental change, populations lag each generation by roughly the same amount the environment is changing. The consequences of this lag are that mortality following selection climbs quickly (Fig. 1c), the number of generations a population is expected to survive drops at faster than a logarithmic pace once lag exceeds even a very small amount (Fig. 1d), and extinction can occur precipitously (Supplementary Figure S2). Variation in the strength of selection $\omega$ and the pace of environmental change, with smaller contributions from heritability and the size of the mutational and equilibrium genetic variances, combine to explain 81% of the variation in the pace of phenotypic change (*R*^*2*^ = 0.81), 66% of the proportion of the population that survive and 75% of the number of generations to extinction (Supplementary Tables S5-S8).

The median population survival time of 141 generations (Table 2) is likely to be an overestimate of what natural populations can achieve: the simulations return populations to carrying capacity each generation (Methods), meaning that extinction only occurs when mortality owing to failure to keep up with the changing environment is so great as to bring about the extinction of the entire population in one generation. But data from 16 Classes of animal plus phytoplankton^34^ suggest that few populations can recover from greater than 50% mortality in a generation (Supplementary Text and Figure S3). When we use this figure as a cut-off point for extinction, the median rate of phenotypic change drops to 0.033 (IQR = 0.017–0.058), and the median number of generations until extinction is 47 (IQR = 25–113, Table 2).

### The maximum sustainable rate of change for a population

A small number of the simulated populations (*n* = 6158 of 119,984, 5.1%) tracked the environment and survived indefinitely (240,000 generations of directional selection). The median rate of environmental change for this group was ${{\Delta_{env/{\sigma_{T}}}}}=0.030738\:$*sds* per generation (IQR = 0.0117–0.0661) and, as expected, the median rate of phenotypic adaptation is only negligibly slower at ${{\Delta_{trait/{\sigma_{T}}}}}$ = 0.030735 (IQR = 0.011–0.066; Table 2). This provides an estimate of the maximum sustainable rate of phenotypic change of approximately 3% of a phenotypic *sd* per generation, or roughly 33 generations to change 1 *sd* and is similar to the value we observe when restricting populations to 50% or more survival.

The value of approximately 3% (IQR $\sim\:$1–6%) is not highly sensitive to the number of generations used to define “indefinite survival”, but the degree of environmental tracking is. The surviving populations lag the optimum (${{\Delta}}_{env/{\sigma}_{T}}-{{\Delta}}_{trait/{\sigma}_{T}}$) by approximately 3 × 10^−6^ *sds* per generation. By comparison, among populations that survived for at least 10,000 generations of directional selection (*n *= 6762) the median ${{\Delta}}_{env/{\sigma}_{T}}=0.0319$ (IQR = 0.012–0.067) and the median ${{\Delta}}_{trait/{\sigma}_{T}}=0.0312$ (IQR = 0.012–0.067), or approximately 0.0007 *sds* of lag per generation, greater than two orders of magnitude larger. The degree of lag might seem small, but as Fig. 1d shows populations that survive 10,000 generations or fewer are in a region of lag per generation in which long-term survivorship falls logarithmically with increasing pace of environmental change.

Based on these analyses, we focus on the populations that tracked environmental change indefinitely (240,000 generations) as an unambiguous definition of sustained adaptation – all other populations will go extinct from lagging behind the optimum. In this context, even the $\sim$3% figure characteristic of this group may give a biased picture of how rapidly a typical natural population can adapt: the surviving populations are not a random subset of the simulated populations and so may differ from typical populations in characteristics related to long-term adaptation. That turns out to be the case. As a group, survivors had an approximately 2-fold greater input of new genetic variance from mutation per generation than the remaining populations, a key factor in the creation of variance for selection to act on: survivors’ median ${\sigma}_{m}^{2}/{\sigma}_{e}^{2}$ = 0.0078 versus 0.0038 for the remaining populations.

To adjust for this difference in mutation rates, we weighted the surviving populations’ rates of phenotypic change by the probability of their mutation scalar (${\sigma}_{m}^{2}/{\sigma}_{e}^{2})$ and numerically integrated over the sample of surviving populations:

1 $$\Delta _{{trait/\sigma _{T} \left( {adjusted} \right)}} = {{\sum p\left( {{{\sigma _{m}^{2} } \mathord{\left/ {\vphantom {{\sigma _{m}^{2} } {\sigma _{e}^{2} }}} \right. \kern-\nulldelimiterspace} {\sigma _{e}^{2} }}} \right)\left( {\Delta _{{trait/\sigma _{T} }} |{{\sigma _{m}^{2} } \mathord{\left/ {\vphantom {{\sigma _{m}^{2} } {\sigma _{e}^{2} }}} \right. \kern-\nulldelimiterspace} {\sigma _{e}^{2} }}} \right)} \mathord{\left/ {\vphantom {{\sum p\left( {{{\sigma _{m}^{2} } \mathord{\left/ {\vphantom {{\sigma _{m}^{2} } {\sigma _{e}^{2} }}} \right. \kern-\nulldelimiterspace} {\sigma _{e}^{2} }}} \right)\left( {\Delta _{{trait/\sigma _{T} }} |{{\sigma _{m}^{2} } \mathord{\left/ {\vphantom {{\sigma _{m}^{2} } {\sigma _{e}^{2} }}} \right. \kern-\nulldelimiterspace} {\sigma _{e}^{2} }}} \right)} {\sum p\left( {\sigma _{m}^{2} /\sigma _{e}^{2} } \right)}}} \right. \kern-\nulldelimiterspace} {\sum p\left( {\sigma _{m}^{2} /\sigma _{e}^{2} } \right)}}$$

where $p({\sigma}_{m}^{2}/{\sigma}_{e}^{2}$) is the probability of observing a given mutation scalar as determined from the ${\sigma}_{m}^{2}/{\sigma}_{e}^{2}$ probability density distribution, and the summation is over all *n* = 6158 surviving populations. The calculation yields a median ${{\Delta}}_{trait/{\sigma}_{T}}\left(adjusted\right)$ = 0.019 (IQR = 0.006–0.055; Table 2). This is the rate of ${{\Delta}}_{trait/{\sigma}_{T}}$ expected for surviving populations with the same input of genetic variance each generation as samples with mutational variances representative of natural populations.

### Theoretical estimate of the sustainable rate of phenotypic change

Quantitative genetic theory^2^ provides an estimate of the maximum rate of environmental change beyond which a population will go extinct, denoted ${k}_{c}$ for the critical (or maximum) rate. Species whose rates of phenotypic adaptation are less than ${k}_{c}$ will be able to sustain those rates (barring extinction from stochastic effects), otherwise they will be on a trajectory to extinction owing to mortality and loss of genetic variance (Supplementary Figure S2). The estimator does not make use of information on the rates of environmental and phenotypic change, or the amount of new variance obtained each generation from mutation. It can be estimated in the present setting from:

2 $$\:{k}_{c}\sim\frac{{\sigma}_{g}^{2}}{{\sigma}_{g}^{2}+{V}_{s}}\sqrt{\left({V}_{s}+{\sigma}_{g}^{2}\right){log}_{e}\left[\frac{{N}_{o}\omega\:}{\sqrt{{V}_{s}+{\sigma}_{g}^{2}}}\right]}$$

where ${\sigma}_{g}^{2}$ is the genetic variance as observed at the mutation-selection balance equilibrium, ${V}_{s}={\omega}^{2}+{\sigma}_{e}^{2}$, and *N*_*o *_= the number of offspring per surviving parent, calculated here as the expected number of offspring under random mating with replacement and given that we return populations to their carrying capacity each generation. The derivation of *k*_*c*_ assumes that mean population fitness is high enough during the period of environmental change that the effective population size is constant^2^.

Estimating ${k}_{c}$ from Eq. 2 within the populations that survive indefinitely (across all population characteristics and selection regimes) and scaling it by the trait *sd* yields a median ${k}_{c}$= 0.022 (IQR = 0.008–0.056; Table 2). Restricting these surviving populations to less than 50% mortality, the median ${k}_{c}$ falls slightly to 0.021 (IQR = 0.008–0.053, Table 2; $\sim$70% of ${k}_{c}$ values $\le$ 0.04). This is pleasingly close to (and appropriately just larger than) the median rate of 0.019 *sds* per generation that we observe in the surviving populations when rates of mutation are adjusted to be representative of natural populations (Table 2, bold).

### Field data reveal comparable rates of phenotypic change

**Fig. 2:**
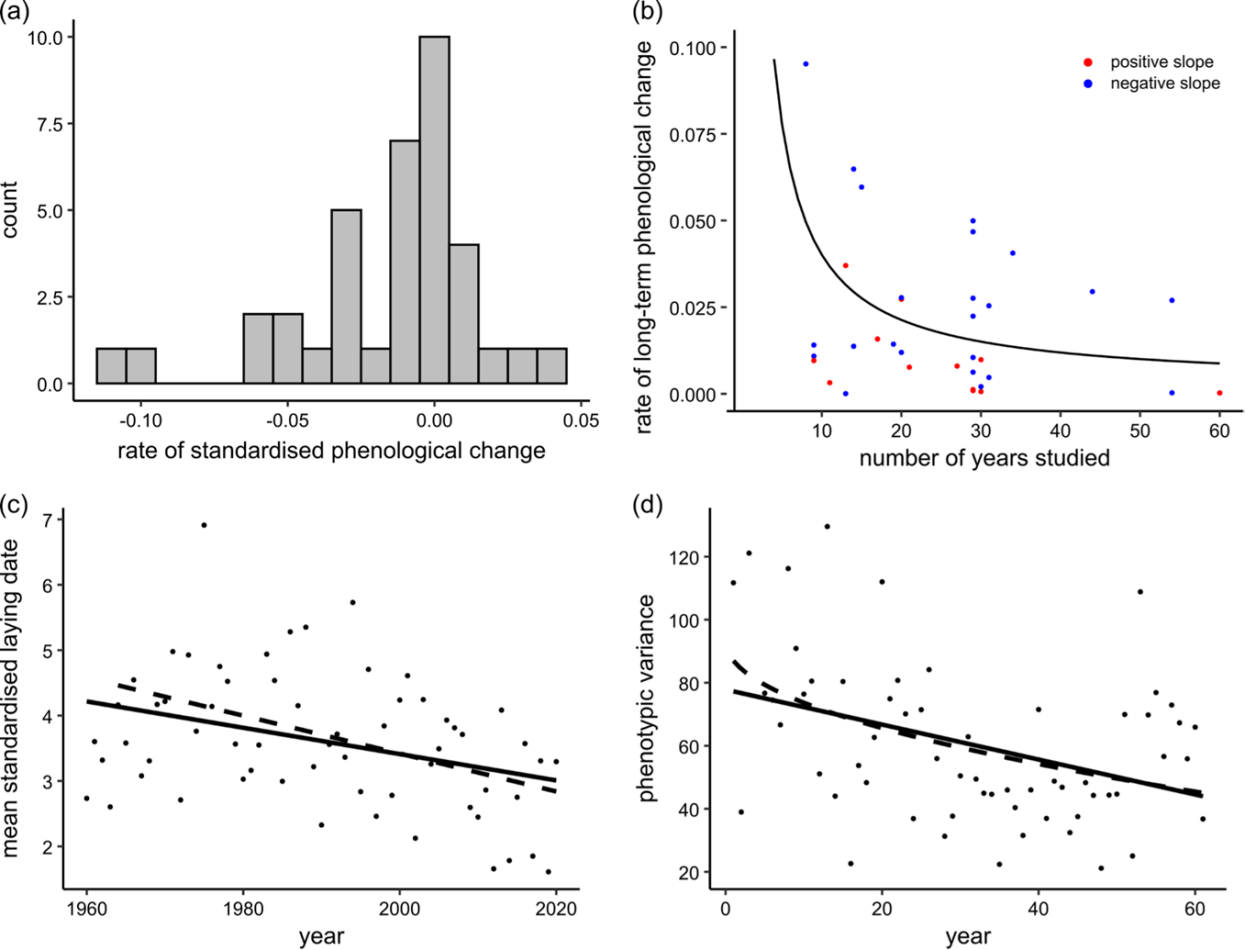
Comparisons to field data. (**a**) distribution of rates of phenological change in standard deviations per annum, data from reference^10^; (**b**) rates of long-term phenological change versus number of years studied. Blue dots are studies with advancing phenologies (*n* = 25, all bird species, some red dots obscure blue dots); (**c**) population mean standardised laying dates by year for Great Tits (*Parus major*), slope for sixty-year period =−0.021, *p* < 0.006; dashed line excludes first four years, slope = −0.029, *p* < 0.0004, data from reference 39 (**d**) population phenotypic variance by year for Great Tit populations, linear effect *p* < 0.0007, years^1/2^ effect *p* < 0.0001 (dashed line). Data from B. Sheldon, pers comm.

Using data available from an extensive and carefully curated meta-analysis^10^ of phenological changes in warming climates (Methods), we calculated the rate at which standardised phenotypic means changed per annum for thirty-seven populations studied for a median of twenty-nine years (Fig. 2a,b, none of these data was used in setting the input parameters of the simulation model). Phenotypic traits included laying dates, arrival times, incubation times and nesting times (all but one of the studies was on birds).

Twenty-five of the thirty-seven (all bird species) show phenology advancing (positive rates, Fig. 2a) and at a median rate of 0.022 standard deviations per annum (IQR = 0.008–0.0436, range ~ 0 to 0.11, *n* = 25). Assuming an average generation length in these species of 2–4 years, a 0.022 change per annum implies a median per generation change of 0.044 to 0.088 standard deviations (i.e., 11 to 23 generations to produce one standard deviation). These fall at the 68th and 86th percentiles respectively of the ${k}_{c}$ distribution. Assuming these changes are genetically based, we might therefore suspect that these species are, on average, already being made vulnerable to environmental changes, and this is consistent with the conclusions of a previous meta-analysis of these populations^10^ that indirectly estimated ${k}_{c}$ for these species. It might also suggest that species with generation times longer than four years will begin to fall outside our plausible ranges.

Individually some of the twenty-five species in Fig. 2a are changing at rates that exceed all but the upper range of phenotypic rates we observe among survivors in our simulations. The most rapidly evolving population, a species of Eurasian owl (*Otus scops*) was studied over just seven years and is advancing its phenology at ~ 0.11 standard deviations per annum or ~ 0.22 standard deviations or more per generation given its approximately two-year generation time, a rate of change that puts it in league with artificial selection studies that artificially maintain population sizes. Some or all of this change might be attributable to phenotypic plasticity (see Discussion for more on phenotypic plasticity), but a changing environment can itself initially increase the genetic variance in fitness^35^. Consequently a possible genetic explanation for the rapidly changing populations in Fig. 2b is that they have not been studied for very long and so may still have reserves of genetic variance to fuel change: the longer the 25 populations with advancing phenology had been studied the slower their rate of phenotypic change (Fig. 2b; as directional series, these points do not suffer from the rate/generation artefact^36^). The curve in Fig. 2b predicts a long-term rate of change around 0.01 *sds* per annum or roughly 0.02 to 0.04 *sds* per generation for most bird species, which falls comfortably in the middle of our predicted maximum sustainable rates.

For comparison, the rate of phenotypic change declines similarly to Fig. 2b with the length of the time a population was studied across 13 datasets that Arnold^37^ (Table 14.1 therein) reports, and the predicted rate of change is 0.015 *sds* per generation for the longest time-series. Hendry and Kinnison^38^ report (their Table 1) a median standardised phenotypic change for 31 populations, including bird and fish species, of 0.055 (IQR = 0.024–0.382) across 1 to 111 generations. The rate of change drops with the number of generations a population was studied (as in Fig. 2b) according to a power law (*r* = 0.88) such that confining the analysis to populations studied greater than twenty generations yields a median rate of change of 0.0185 (IQR = 0.007–0.038; *n* = 12), very similar to the distribution of ${k}_{c}$ (Table 2).

A 60-year study^39^ within a single species is consistent with the 0.02–0.04 range and the suggestion that they could be under strong selection. Great Tits’ (*Parus major*) mean laying dates in the Spring have been advancing since 1960 and at a rate of 0.021 *sds* per annum (Fig. 2c; see also an earlier study from this group^40^). Their advancing phenologies are thought to be in response to increasing mean temperatures and the availability of caterpillars, which are appearing earlier in the season^40^. Generation length is around two years in Great Tits, suggesting these birds might need to lay their eggs 0.04 to 0.06 *sds* earlier per generation, values that fall in the 65th to 76th percentiles of the surviving populations’ ${k}_{c}$ distribution (an earlier modelling study estimated ${k}_{c}$ indirectly for this Great Tit population^41^ and concluded they could survive “mild” rates of climate change but that higher rates of change would exceed their capacities to adapt). Within-population variances in the 60-year study have declined roughly 50% over time (Fig. 2d), and possibly more rapidly at first, perhaps suggesting they are under strong selection. Alternatively, the authors of the 60-year field study^39,40,42^ suggest that phenotypic plasticity can account for the trend of phenotypic changes observed so far (and see Discussion). An analysis^43^ with parameter values tailored to Schneider’s toad (*Rhinella diptycha*) estimated the mean ${k}_{c}$ at 0.037 ± 0.009, a value that falls at the 66th percentile of the ${k}_{c}$ distribution in Table 2.

## Discussion

Theoretical estimates of the maximum rate of sustainable phenotypic change and the field data we present are consistent with our findings that species’ genetic capacities to respond to a changing climate may be largely confined to $\le$2–4% of a trait *sd* per generation. Applying the 2–4% range, keeping up with climate change at the rates observed in the field data discussed in the previous section might not yet pose serious problems for species with one or more generations per annum, at least on average. But for many longer-lived and typically more slowly reproducing species, phenotypic adaptation might prove a challenge. We estimate this to include most species with generation times greater than around three to four years, and even those with generation times of two to four years might already be falling behind environmental change. A species such as the polar bear (*Ursus maritimus*) has shown great adaptability^44^, but climate-change-induced loss of sea ice is asking it rapidly to acquire the capability to swim long-ranges without respite^45^ – a tall order for a species with an 11-year generation time.

Evidence increasingly points to the Earth’s warming climate outstripping many species’ capacities to adapt. One recent estimate suggests somewhere between 14 and 32% of macroscopic species could face extinction in the next 50 years^46^. Tree phenological responses are beginning to slow^47^ and tropical forests are failing to track environmental changes^48^. A survey of plants, arthropods and birds in the Arctic, where conditions may be changing most rapidly, found hints that some taxa may have reached the limits of their phenological responses^49^ and advanced egg-laying dates in some bird species are associated with increased chick mortality^50^. Given that the climate has been changing directionally for at least the past 60 years by amounts sufficient to produce measurable effects on species’ phenologies, many species’ capacities for sustained adaptation may already be substantially diminished or perilously trailing behind environmental optima.

Several features of our simulations may affect the generality of our conclusions. We do not model epistasis or dominance. Crow^16^, Hill and colleagues^51^, and Barton and Keightley^14^ suggest that most of the relevant genetic variance will be additive, diminishing the roles of these two factors. That, and the distributional normality of many quantitative traits is consistent with additivity of effects. We have modelled a constant rate of environmental change. It has previously been shown that random environmental variation around a directionally changing optimum generally reduces population survival time and especially when directional selection is weak and environmental variation is large^2^. The role of climatic events could be modelled by drawing environmental changes from fat-tailed distributions such as the Levy or stable distributions (of which the Levy is a special case). We have not studied migration. Local migrant populations will be undergoing similar degrees of directional selection and so may not bring substantial new genetic variance, or even have diminished variance from selection^52^. Migration along, for example, latitudinal climate gradients might mean that migrants simply replace local populations^53^ but do so having become extinct in their previous habitat.

By modelling one trait we have assumed that directional change in the phenotype can proceed indefinitely, unhindered by correlations between traits under selection and other traits of the organism. In real biological settings, correlations among phenotypic traits will limit how much and at what rate species can change and still retain fit phenotypes. We do not suggest that any species could adapt at 2% per generation for 240,000 generations, although, experiments with maize^13^ and with *Drosophila*^11,12^ have produced 10–27 or more standard deviations of change, with phenotypes remaining fit so long as selection is maintained. But this misses the point that most species confronted with a sustained directionally changing environment are not expected to live long – the median time to extinction across the conditions we analysed was 141 generations, and this dropped to 47 when we restricted populations to having 50% or more survival each generation.

We simulated populations of size *n* = 1000. Bürger and Lynch^2^ suggest that when rates of environmental change are high (approaching *k*_*c*_), population size has little effect on the risk of extinction. But when the environment changes slowly, larger populations may have a much lower risk of extinction because stochastic processes (e.g., neutral or genetic drift) are diminished. Species with very large population sizes tend to be those with short generation times (> 1 or even > > 1 per annum) and so, as mentioned, may be at much less risk of a changing climate anyway. We have not investigated systems with a small number of loci of large effect. Our preliminary work (Supplementary Table S4) suggests that rates of phenotypic change slow when the number of loci falls below approximately 10–20 and this makes populations more vulnerable to extinction. Large-scale GWAS’ such as have been brought to bear on other quantitative traits will be required to determine how widespread or representative these “large-effect loci” systems are.

We have not included phenotypic plasticity in our analyses. While it can promote responses to a changing climate, a general model of its genetic architecture and expression^54^ assumes that the gene by environment interaction (the plastic response) takes the form of a linear reaction norm. The model compares the slope of the phenotypic response to environmental change (denoted *b*) to the slope of how the phenotypic optimum changes with the environment (denoted *B*), assumes *b* is invariant across individuals with different genotypes, remains so under sustained directional selection and in extreme environments, and is without cost. When the phenotypic and phenotypic-optimum slopes are similar (as suggested for Great Tits^40,42^) the predicted maximum rates of environmental change can be remarkable: Great Tit laying dates are predicted to be capable of accommodating a nearly 0.5$^\circ$C per annum increase in temperature^42^, and the Schneider’s toad (*Rhinella diptycha*^43^) is predicted to be able to manage a 1$^\circ$C annual increase. These estimates are 25 to 50 times the current global mean rate of warming of $\sim$0.02$^\circ$C per annum and greatly exceed rates of change observed in artificial selection studies.

On the other hand, it is a feature of this model that plasticity’s effect on *k*_*c*_ only really begins to be substantial as *b → B*^43,^ and the model predicts intermediate values of *k*_*c*_ when there are costs to plasticity^54^. For example, the maximum rate of increase in temperature for Great Tits is predicted to fall to 0.028°C per annum when *b* = 0^42^. Although the existence of phenotypic plasticity in promoting phenotypically (facultatively) adaptive responses is not in doubt^55,56^, the relationship between *b* and *B* is seldom known or reported. Chevin et al.^54^ comment that “more information is needed about plasticity and its inheritance in extreme environments before the evolution of plasticity can be included in the analysis of persistence under sustained environmental change”.

With the current state of understanding, the presence and effects of phenotypic plasticity need to be examined on a case-by-case basis (e.g.,^40,42,43^) and although plasticity can buy time it cannot be assumed that it will always ride to the “evolutionary rescue”. In a review of avian plastic responses, Charmantier^57^ concludes (p15) “while plasticity seems common and often adaptive, no study so far has provided direct evidence for an evolutionary [i.e., genetic] response of bird phenology to current climate change”. Kooyers et al.^55^ discussing plants agree, saying (p481), “While plasticity is consistently observed among all focal species, plasticity alone does not seem sufficient to allow adjustment to the multitude of factors changing during climate change.” Arnold et al. conclude that evidence for directional selection acting on [genes for] phenotypic plasticity itself is “sparse”^58^, and in some instances plasticity can impede an adaptive response, such as reported for the response to a temperature cline among seasonally adapted butterflies^59^ (and see also ref 55 for examples in plants such as *Clarkia xantiana*, the Gunsight Clarkia). These considerations make estimates of the expected genetic response to directional change a relevant ‘yardstick’ for a consideration of species’ capacities and vulnerabilities, although it must be allowed that in any given circumstance, particular features of genes, modifiers, and mutational variances could conspire to produce populations capable of adapting at higher, or being limited to lower, rates than we typically find.

On top of affecting individual species, climate change alters entire ecosystems by restructuring the complex and fragile webs of relationships among species that allows the system to be stable. Each of these interactions potentially represents an agent of selection that itself will be influenced by, for example, a changing climate. Robert May^60^ showed over 50 years ago that for ecosystems to have a high probability of being stable against perturbations in species’ population sizes, the average influence species have on each other must be small and grow smaller roughly proportional to ${a}_{ij}<1/{n}^{1/2}$, where ${a}_{ij}$ is the influence of species *i* on species *j* and $n$ is the number of species in the ecosystem: large stable ecosystems require that most interactions are weak. May’s analytical results generalise simulation studies^61^ and have been verified in meta-analyses of large complex ecosystems^62^.

May noted that the transition from a stable to an unstable ecosystem linked to changes in the ${a}_{ij}\:$is “very sharp”, and especially so the larger the number of species. With many interacting species, the web of direct and indirect influences is so complex as to defy simple intuitions about the effects of even small changes to the ${a}_{ij}$. The significance of this for climate change studies is that a directionally changing climate will mean some species’ population sizes dwindling, with knock-on effects on the strengths of their interactions (the size of the ${a}_{ij}$) with other species – interactions that in many cases will be required for survival, such as among predators and prey or food sources more generally. This could provide an explanation for why shifts in ecosystems are often catastrophic rather than gradual^63^. Or as Kareiva^32^ has remarked more trenchantly, ‘‘It would not be surprising to see entire patterns of community organization jumbled as a result of global change’’.

Generalising methods such as we have used here to examine species adapting within ecosystems that comprise sets of species with differing responses to a changing climate and varying degrees of connection could yield important insights. For example, under the influence of a directionally changing climate we might expect to see ecosystems evolving toward smaller sets of species which do not or only weakly rely on each other for survival. Such reduced ecosystems could emerge naturally as the survivors in a newly unpredictable or extreme world.

## Methods

### The simulation model

We simulate the evolution of a quantitative trait evolving under stabilising and directional selection in a population of *n* diploid individuals. An individual’s genotypic trait value is given by the sum of the effects of the alleles within and then over the *k* loci that comprise its genome, with no dominance or epistasis, yielding a genotypic variance across individuals denoted by ${\sigma}_{g}^{2}$. We use *k* = 100, 500, 1000, and 5000 loci reflecting the recognition that large numbers of loci may affect quantitative traits such as body size and life histories^21^. Because loci may often follow something like an exponential distribution in their effects on quantitative traits^23,^ our use of *k* equivalent loci may mimic the behaviour of a larger number.

The phenotype is obtained by adding a random environmental element with a mean of zero and variance ${\sigma}_{e}^{2}$ to the genotypic value. The environmental effects are equally likely to increase or decrease the value of the trait, and the expected mean genotype is the same as the expected mean phenotype. The population variance in the quantitative trait, ${\sigma}_{T}^{2}$, is then the sum of the genetic (${\sigma}_{g}^{2}$) and environmental (${\sigma}_{e}^{2}$) variances across the *n* individuals: ${\sigma}_{T}^{2}={\sigma}_{g}^{2}+{\sigma}_{e}^{2}$.

### Selection

We apply selection to the phenotypic values of the individuals within a population via a Gaussian fitness function with some optimal value $\theta$ and specified standard deviation of fitness, ${\sigma}_{\omega}$, where the parameter $\omega$ corresponds to the parameter used in the simulations. Other things equal, smaller standard deviations of the fitness function produce stronger selection within populations and vice versa by altering the rate at which fitness declines away from the current optimum. We scale the Gaussian function so that phenotypes at the optimum have a fitness of 1. An individual’s probability of survival is given by their fitness and modelled as a coin-toss with a probability of ‘heads’ (survive) equal to the fitness of the trait. This means that any phenotype can survive but some are more likely to than others.

### Reproduction

To create the next generation, survivors are sampled with replacement and then undergo mating between random pairs. Each parent produces two haploid gametes with mutation and with recombination among alleles (below). An offspring’s genetic component of the trait is assembled from the two randomly chosen gametes (parents cannot mate with themselves). The genotypic value is augmented by a component of random mutation drawn from a distribution with a mean of zero and a variance of ${\sigma}_{m}^{2}$ (see below). Finally, a new random environmental component is added to yield ${\sigma}_{T}^{2}={\sigma}_{g}^{2}+{\sigma}_{m}^{2}+{\sigma}_{e}^{2}$. This random mating process is repeated until the population is returned to *n* individuals, meaning some survivors by chance produce more offspring than others, with recombination and mutation occurring independently every time a parent produces gametes. Mutated loci are then inherited in the offspring of survivors.

### Mutation

The mutational model draws on prior theoretical work developed to understand the long-term persistence of populations undergoing directional selection^2,64–66^. The probability that an allele at a locus mutates is ${\mu}_{m}=1\times{10}^{-4}$. Loci are assumed to comprise many nucleotides whose individual mutation rates would be far lower. The mutation rate is fixed across loci unlike in some models in which ${\mu}_{m}$ is allowed to vary^67,68^. We draw the number of mutations per haploid genome from a *Poisson* distribution with mean $k{\mu}_{m}$. The loci chosen to mutate are picked at random (typically no more than one locus mutates). This process is repeated independently in both haploid genomes, yielding a genomic mutation rate of 2$k{\mu}_{m}$.

Allelic effects (the effect on the phenotype of a mutation at a locus) in mutated loci are drawn from a Gaussian distribution with mean = 0 and variance given by ${m}^{2}$, where ${m}^{2}$ is chosen such that $2k\mathrm{\mu}_{m}m^{2} = \sigma^{2}_{m}$, where ${\sigma}_{m}^{2}$ is the new variance added by mutation. A common observation is that the mutational variance is on the order of *10*^*−2*^ to *10*^*−4*^ of the environmental variance ${\sigma}_{e}^{2}$^18,27,30,31,69^. Measurements of ${\sigma}_{m}^{2}/{\sigma}_{e}^{2}$ ratios are available from the literature (below) and thereby provide a probability distribution of ‘mutation scalars’ that guide the choice of ${m}^{2}$.

For a given ${\sigma}_{m}^{2}$, when the number of loci (*k*) is small, there are fewer mutations but each one is of larger individual variance (${m}^{2}$), and vice versa. This framework ensures that our simulations have the same expected ${\sigma}_{m}^{2}$ for differing numbers of loci and is consistent with the estimation of mutational variances in laboratory studies where the number of loci that affect the phenotypic trait is rarely known. Drawing allelic effects from a continuous distribution simulates the continuum-of-alleles model^70^, which can permit more change in the limit than models with a finite number of alleles^67^.

### Recombination

Recombination events between each parent’s two haploid genomes are drawn from a Poisson distribution with mean ${\mu}_{r}$. In preliminary work, we found that values of recombination ${\mu}_{r}$ = 3 (90% range 1–5 events per haploid genome) or 5 (90% range = 2–8) yielded the largest genetic variances and rates of phenotypic change under directional selection and mimic a quantitative trait whose loci are distributed among several (or more) chromosomes. Larger values of ${\mu}_{r}$ tended to homogenise genomes; smaller values evidently yielded fewer novel allele combinations and smaller genetic variances.

### Stabilising and directional selection

Under stabilising selection, the optimal value of the trait remains constant at zero, and we assess phenotypes against the Gaussian fitness distribution centred at zero and of width (standard deviation) $\omega$. To produce directional selection, we move the optimum value of the trait (the mean of the fitness function) by a fixed amount ${{\Delta}}_{env}\:$each generation independently of the population’s response.

Simulations run for 10,000 generations of stabilising selection (${{\Delta}}_{env}$ = 0), followed by directional selection ${({\Delta}}_{env}>0)$ until the population goes extinct or up to a maximum of 250,000 generations (10,000 stabilising generations plus 240,000 of directional selection). The upper limit of 250,000 generations was set based on preliminary work showing that populations surviving 240,000 generations of directional selection were tracking ${{\Delta}}_{env}$ whilst those surviving fewer generations were falling behind and would therefore go extinct; although in the long run any population subjected to the effects of drift will eventually go extinct.

### Characterisation of the simulated populations

We randomly generated 15,000 populations of size *n* = 1000 for each of the four categories of numbers of loci by two different rates of recombination yielding 120,000 simulated populations. Populations were randomly assigned a set of fixed starting conditions by independently sampling values from the probability distributions of five input variables: the within-population variance of the phenotypic trait in natural populations (${\sigma}_{T}^{2}$), heritability of the trait (*h*^*2*^, broad sense, *h*^*2*^$\:={\sigma}_{g}^{2}/{\sigma}_{T}^{2}$), the strength of within-population selection ($\omega$), the amount by which the environmental optimum changed every generation during the period of directional selection, and finally the amount of new variance in the phenotypic trait arising each generation owing to mutation $({\sigma}_{m}^{2})$.

We derived the probability distributions of the phenotypic trait, heritability, and the size of the mutational variance relative to the environmental variance, ${\sigma}_{m}^{2}/{\sigma}_{e}^{2}$ – hereafter called the mutational scalar – from published meta-analyses^6,17,27,28^ (see below). The width of the fitness distribution within populations $\omega$, although fixed for any given population, was varied uniformly from 0 to 10 across populations, and ${{\Delta}}_{env}$ ~ *U(0–0.3)* in a similar fashion; from preliminary testing, we found that these two ranges generated a distribution of strengths of directional selection comparable to those observed in empirical meta-analyses as described below.

We generated the starting conditions of the 120,000 simulated populations by repeatedly randomly sampling from the distributions of the five input variables. The values of the input variables are fixed for the entire simulation yielding a set of populations that numerically integrates over a space of possibilities $({\sigma}_{T}^{2}$ $\times$ *h*^*2*^ $\times\:\omega\:\times$ ${\sigma}_{m}^{2}/{\sigma}_{e}^{2}\times{{\Delta}}_{env})$ that might be observed in nature.

### Probability densities of model parameters

We derived the probability densities of the model’s parameters that we used in the simulations from published meta-analyses, as described below.

#### Phenotypic variance

Hansen and Pelabon^17^ report *n* = 1539 phenotypic trait variances from field studies with values suitable for converting to an estimate of the variance on a logarithmic scale (to remove scale dependency). We converted these variances using the approximation ${\sigma}^{2}(log(x) ~ [CV(x)]^{2})$, which is valid when $CV\left(x\right)\le0.3$^71^ where $CV\left(x\right)$ is the coefficient of variation of the untransformed data. Data source: 10.1146/annurev-ecolsys-011121-021241.

Several observations from the larger dataset had estimated heritabilities of traits less than 0 or greater than 1, or estimated additive variances less than 0, and these were excluded. The distribution of *n* = 1539 phenotypic variances is approximately exponentially distributed with a mean of 0.017 and a range of ~ 0–0.09. Controlling for trait name (same trait in different studies) yields 499 observations with a mean of 0.022. Their weighted average is ~ 0.018.

We also extracted within-population phenotypic means and variances for traits reported in studies cited in a meta-analysis of directional selection in natural populations^6,28^. Data source: (10.5061/dryad.7996). This yielded 1087 estimates of variance suitable for conversion to a log-scale (as above). As above, these were also approximately exponentially distributed, and with a mean of 0.020 and a range of ~ 0–0.09. Controlling for trait name yields *n* = 956 observations with a mean of 0.022.

Based on these empirical distributions, we drew input variances in the simulations randomly from an Exponential (0.018) distribution excluding draws < 0.01 to reduce ‘basement’ effects that might arise from neutral or genetic drift for very small variances and with an upper limit of 0.09. This returns a distribution with a median of about 0.022.

#### Heritability

Hansen and Pelabon^17^ report *n* = 2327 heritability estimates on 169 species drawn from ten biological Classes. The distribution of these heritabilities is described by a Weibull (shape = 1.6, scale = 0.4) distribution. Controlling for trait name yields *n* = 890 estimates with Weibull parameters shape = 1.53 and scale = 0.37. Data source: 10.1146/annurev-ecolsys-011121-021241. We drew heritabilities in the simulations from a Weibull (1.6, 0.4).

####  Mutational “heritabilities” ${\sigma}_{m}^{2}/{\sigma}_{e}^{2}$

A rule-of-thumb in quantitative genetics beginning with Alan Robertson in the 1950s^29^ is that the mutational variance is on the order of 10^−3^ of the environmental variance (i.e., ${{\upsigma}}_{\text{m}}^{2}/{{\upsigma}}_{\text{e}}^{2}\sim{10}^{-3}$). More recent data suggests mutational variances typically fall in the range of 10^−2^ to 10^−4^ times the environmental variance^27,30,31^. A carefully curated meta-analysis of published studies reporting 163 mutational heritabilities (${\sigma}_{m}^{2}/{\sigma}_{e}^{2}$) from plants and animals^27^ yields an approximately exponential distribution with a median of $2.2\times{10}^{-3}$ (controlling for species) and 95% range from $6\times{10}^{-5}$ to $1.8\times{10}^{-2}$. We get nearly identical results weighting the observations by the inverse of the standard error of the mutational heritability estimate. Data source: 10.6084/m9.figshare.14913051.

We drew mutational heritabilities randomly from a Gamma (1.1, 0.004) yielding a mean of ~ 0.0044 and 95% interior range of ~ 0.0002 to 0.019. We used the Gamma because it is very similar to the exponential for these parameter values, but unlike the exponential distribution it has a mode, whereas the exponential does not. We prefer this distribution on the assumption that a mutation rate of zero is not the most frequently occurring in nature (as implied by an exponential fit). This creates a prior distribution on the mutational variance that has nearly all its area in the range of 10^−2^ to 10^−4^ of the environmental variance.

For comparison, mutational variances for 56 traits in plants and animals reported by Houle et al.^31^ are well-characterised by a log-normal curve with a geometric mean of 0.0021 and a 95% range between 0.0001 and 0.03.

#### The strength of directional selection

The strength of directional selection, measured as the change in a proxy for fitness (such as litter size or clutch mass) for a one standard deviation change in the phenotypic trait (the standardised selection coefficient), is available for a sample of *n* = 2720 populations (we used only those studies that reported a slope > 0). Data source: https://doi.org/10.5061/dryad.7996^6,28^. Selection on fitness itself would yield a slope of 1. Scaling the estimates of the proxies for fitness by their 99.5th percentile to yield a maximum of 1 provides a distribution well-characterised by a log-normal curve with *u* = −2.56 and *σ* = 1.19. We randomly drew the strength of directional selection parameters used in the simulations from this distribution.

### Field studies of phenological change

The data used in Fig. 2a,b are available in tabular form from Radchuk et al.^10^: https://www.nature.com/articles/s41467-019-10924-4#MOESM8.

The data on laying dates used in Fig. 2c are available from Cole et al.^39^: 10.6084/m9.figshare.14345960.v1.

## Supplementary Information

Below is the link to the electronic supplementary material.

Supplementary Material 1

## Data Availability

The code used to generate the simulations, raw data output, and data for the Figures is available at http://www.evolution.reading.ac.uk/QuantitativeGenetic/Repository.zip.
