# Supplementary material for: Quantitative-genetic analysis of directional adaptation suggests low maximum sustainable rates of change in agreement with data from field populations
Source: Sci Rep. 2025 Dec 3;15:43116. doi: 10.1038/s41598-025-24445-2 (PMC12678822; doi:10.1038/s41598-025-24445-2)
Supplement: Supplementary file 1 — Supplementary Material 1 [file 41598_2025_24445_MOESM1_ESM.docx]

**Supplementary Information**

Quantitative-genetic analysis of directional adaptation suggests low maximum sustainable rates of change in agreement with data from field populations

Mark Pagel^1*^, Jacob D. Gardner^1^ and Andrew Meade^1^

School of Biological Sciences,

University of Reading,

Reading, UK

*author for correspondence

Mark Pagel

Email: [m.pagel@reading.ac.uk](mailto:m.pagel@reading.ac.uk)

**Supplementary Text**

Summary of simulation design

Step 1. Choose a value of *k*, the number of loci (*k* = 100, 500, 1000, 5000). Initiate a population by drawing a random value of the phenotypic variance, heritability, mutation scalar (${\sigma_{m}^{2}}/{\sigma_{E}^{2}})$, width of the fitness function, and the rate of environmental change per generation ($\Delta_{\mathrm{env}}$) from the five probability densities. These are fixed for the simulation. Generate a population of size n = 1000 from the first four characteristics. Each individual in the population has a diploid genome of *k* loci with initial values equal to zero.

Step 2. Allow populations to evolve under stabilising selection ($\Delta_{\mathrm{env}}=0$) for the first 10,000 generations, adding a random component of mutational variance $\sigma_{m}^{2}$ plus a new environmental component $\sigma_{e}^{2}$ every generation. Loci accumulate changes on the genetic component of the phenotype, which are inherited following selection, but not the environmental component which is newly assigned every generation. Introduce directional selection ($\Delta_{\mathrm{env}}>0$) at generation 10,000, and continue until the population goes extinct or reaches a maximum of 250,000 generations.

Step 3. Record outcome variables per generation, including the current phenotypic mean and variance, genetic variance, the strength of selection on phenotypes, survivorship, and average phenotypic change from the previous generation.

Step 4. Repeat steps 1–3 15,000 times for each of the eight $k\times\mu_{r}$ ($\mu_{r}=3, \mu_{r}=5$) combinations.

Step 5. Repeat steps 1–4 separately for ‘genomes’ of 100, 500, 1000, and 5000 loci, for a total of 120,000 simulated populations.

Posterior summary of the data: create a summary data file of 120,000 rows (one for each simulated population) containing information on the input parameters and the summary statistics describing genotypic and phenotypic means and variances, rates of change, strengths of selection, and mortality rates.

Preliminary results of the model and simulations

We checked the model’s performance against theoretical predictions and examined whether it yielded strengths of directional selection comparable to those observed in field studies of natural populations. Bürger and colleagues^1,2^ derived an analytical estimate of the genotypic variance of a polygenic trait in mutation-selection balance whilst undergoing stabilising selection for a fixed optimum. Labelled the stochastic-house-of-cards estimator, it is given by

$$\sigma_{g(SHC)}^{2}={4nu(\omega^{2}+\sigma_{e}^{2})}/{(1+\frac{(\omega^{2}+\sigma_{e}^{2}}{N_{e}m^{2}}})$$

where we estimate the effective population size *N_e_* from Falconer’s^3^ equation 4.7 and assuming a binomial distribution of reproductive success given that mating was random; the values of the remaining parameters are as previously defined and are fixed at the start of each simulation. We calculated $\sigma_{g(SHC)}^{2}$ for each of our simulated populations based upon their input conditions and correlated it with the observed within-population genetic variances measured at the end of the period of stabilising selection. The correlation across all populations and separately for differing numbers of loci are high and the slopes are all close to 1.0 (Figure S1a). Levels of recombination had a negligible effect (Figure S1 caption).

**
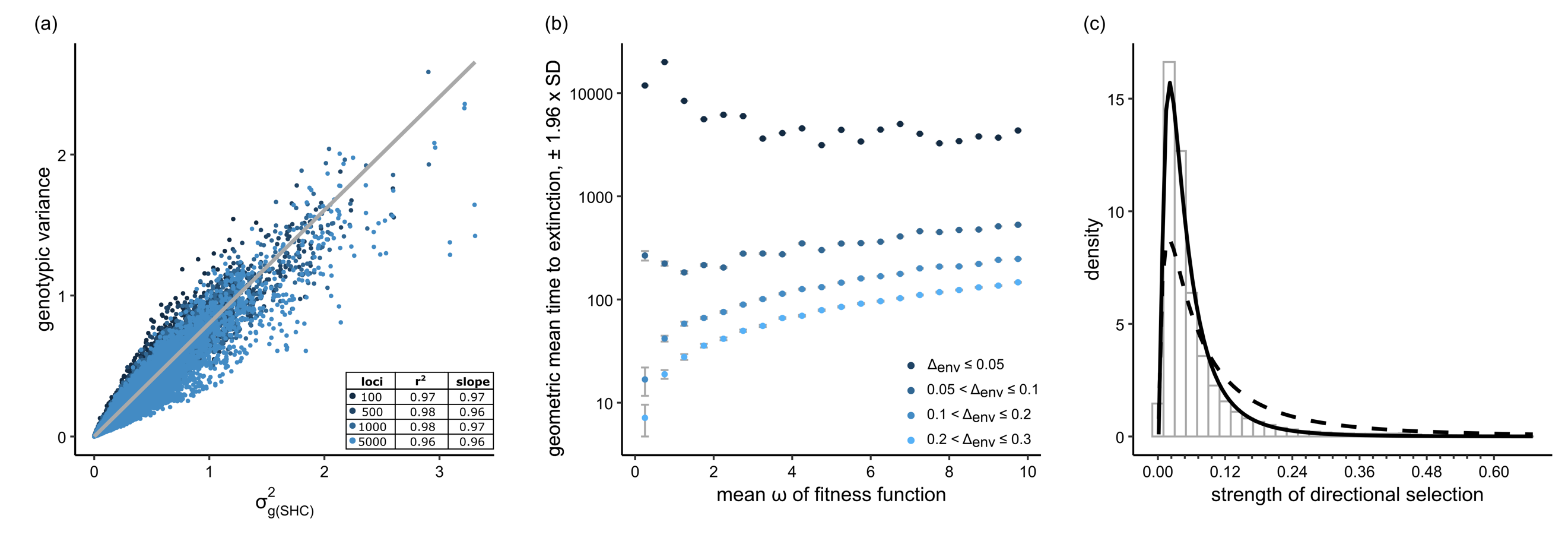
**

**Figure S1. Preliminary simulation outcomes.** a) mean genetic variances for the 119,984 simulated populations at the end of the period of stabilising selection versus predicted genetic variances at mutation-selection equilibrium (Bürger et al.^2^, *r^2^* = 0.97, slope = 0.96 ± 0.0005, log-log transformed to normalise variance. Inset shows results for differing numbers of loci. Level of recombination had a negligible effect: for $\mu_{r}=3,5$, slopes = 0.966, 0.956 ; b) geometric mean number of generations ± 1.96 $\times$ standard deviation to population extinction as a function of the strength of selection ($\omega$, divivded into 20 bins) for four levels of $\Delta_{\mathrm{env}}$: from top $\Delta_{\mathrm{env}}\leq$ 0.05, 0.05 > $\Delta_{\mathrm{env}}\leq$ 0.1, 0.1 > $\Delta_{\mathrm{env}}\leq$ 0.2, and 0.2 > $\Delta_{\mathrm{env}}\leq$ 3 ($\Delta_{\mathrm{env}}$ not scaled by phenotypic trait *sd*); c) frequency distribution of strength of directional selection measured as the change in fitness for a 1 s*d* change in the phenotype at the generation a population reached 50% mortality (solid fitted log-normal curve: $\mu=-3.16, \sigma=0.85$) versus log-normal dashed curve: $\mu=-2.56, \sigma=1.19$ from meta-analysis of published data (main text, Methods).

It has been noted^1,4^ that for low rates of environmental change $\Delta_{env}$, the time (generations) it takes a population to go extinct when adapting to a constantly moving environmental optimum, has an intermediate maximum when plotted against the strength of within-population selection $\omega$. We observe this intermediate maximum for $\Delta_{env}\leq0.05$ at $\omega=0.75$ (Figure S1b). The effect is suggested to arise^1^ because as the fitness distributions becomes narrower ($\omega\to0)$ and within-population selection becomes stronger, genetic variance is depleted and populations cannot respond to selection, whereas for very weak selection ($\omega\gg1)$and a low rate of environmental change populations become increasingly governed by drift. The intermediate maximum disappears for higher rates of $\Delta_{env}$, with time to extinction now generally increasing with $\omega$ (Figure S1b).

We calculated the change in fitness (as read off the Gaussian fitness curve) within each of our simulated populations for a one standard deviation change in the phenotype, measured during the period of directional selection when population survivorship was at 50% of the starting population size of 1000. This is a measure of the realised strength of directional selection. The distribution of these standardised slopes across the 119,984 simulated populations (Figure S1c) is in broad agreement with the distribution of the same measure as reported from field studies^5^ and from a meta-analysis of over 2000 traits undergoing short-term directional selection in the wild^6,7^.

**Table S1.** Generations to 50% population mortality and to extinction by number of loci. Table reports medians and Interquartile Ranges (IQR) as outcomes are skewed.

| No. loci | All | 100 | 500 | 1000 | 5000 |
| --- | --- | --- | --- | --- | --- |
| Generations to 50% mortality, median (IQR) | 47  **(25–113)** | 45  (24–108) | 47  (25–114) | 47  (25–115) | 47  (25–116) |
| Generations to extinction, median (IQR) | **141**  **(79–326)** | 139  (76–328) | 141  (79–329) | 142  (79–327) | 142  (80–323) |

Values for $\mu_{r}=3,5$: $\mu_{r}=3$ generally > $\mu_{r}=5$ by 1–2 generations.

**Table S2.** Genetic variances and strengths of within-population selection. Table reports medians and Interquartile Ranges (IQR) as outcomes are skewed.

| No loci | Genetic variance at  generation 10k,  median (IQR) | Genetic variance at  50% mortality,  median (IQR) | Surviving standardised slope at 50% mortality, median (IQR) |
| --- | --- | --- | --- |
| All | 0.0785 (0.031–0.169) | 0.0701 (0.029–0.146) | 0.039 (0.023–0.071) |
| 100 | 0.0658 (0.025–0.143) | 0.0617 (0.025–0.131) | 0.038 (0.023–0.067) |
| 500 | 0.080 (0.032–0.173) | 0.072 (0.029–0.149) | 0.040 (0.023–0.072) |
| 1000 | 0.083 (0.033–0.179) | 0.073 (0.031–0.153) | 0.040 (0.024–0.073) |
| 5000 | 0.086 (0.034–0.184) | 0.074 (0.031–0.155) | 0.040 (0.024–0.074) |

Genetic variances: Values for $\mu_{r}$ $=3$ generally > $\mu_{r}=5$ by 0.001–0.003 at generation 10k and by 0–0.005 at 50% mortality; slopes differ negligibly.

**Table S3.** Rates of phenotypic change in trait *sds* per generation. Table reports medians and Interquartile Ranges (IQR) as outcomes are skewed.

| No loci | Standardised phenotypic change per generation to 50% mortality, median (IQR) | Standardised phenotypic change per generation to extinction, median (IQR) |
| --- | --- | --- |
| All | 0.033 (0.017–0.059) | 0.056 (0.030–0.103) |
| 100 | 0.030 (0.016–0.051) | 0.053 (0.029–0.096) |
| 500 | 0.033 (0.017–0.060) | 0.057 (0.030–0.104) |
| 1000 | 0.033 (0.017–0.061) | 0.057 (0.031–0.107) |
| 5000 | 0.034 (0.017–0.063) | 0.058 (0.031–0.108) |

Median phenotypic change for $\mu_{r}$ $=3$ generally > $\mu_{r}=5$ by 0–0.001 for 50% mortality and by 0–0.003 for extinction.

**Table S4.** Generations to extinction and rates of phenotypic change in trait *sds* per generation for populations with 1–100 loci (based on preliminary investigations).

| No loci | Generations to extinction,  median (IQR) | $\boldsymbol{\Delta}_{\boldsymbol{trait}}\boldsymbol{,}$sds per generation,  median (IQR) |
| --- | --- | --- |
| 1 | 123 (65–266) | 0.015 (0.0007–0.061) |
| 5 | 129 (70–295) | 0.036 (0.016–0.077) |
| 10 | 132 (72–309) | 0.042 (0.021–0.084) |
| 20 | 136 (73–323) | 0.047 (0.025–0.087) |
| 50 | 138 (75–329) | 0.052 (0.028–0.093) |
| 100 | 139 (76–332) | 0.055 (0.030–0.098) |
| 100–5000  (from main text Table 2) | **141 (79–326)** | **0.056 (0.030–0.103)** |

Variation in time to extinction and in the pace of phenotypic change

**Table S5.** Correlations of selected variables with population survival per generation, per generation rate of phenotypic change, and the number of generations to extinction^a^.

| Variable^b^ | Percent population survival | Standardised phenotypic change per generation  (sd units) | Number of  generations to extinction |
| --- | --- | --- | --- |
| Heritability, *h^2^* | 0.0155 | 0.0742 | 0.0489 |
| Equilibrium $\boldsymbol{\sigma}_{\boldsymbol{g}}^{\mathbf{2}}$ | 0.1500 | 0.2951 | 0.245 |
| $\boldsymbol{\sigma}_{\boldsymbol{m}}^{\mathbf{2}}$ | 0.1127 | 0.4950 | 0.224 |
| Environmental change per generation | -0.6831 | 0.2540 | -0.837 |
| Strength of selection around optimum (-$\boldsymbol{\omega}$) | -0.4128^c^ | 0.5910 | -0.239 |

Notes: a) variables are logarithmically transformed where it improves the linear relationship. All correlations significant at p < 0.0001 owing to large sample size (n = 119,984, see main text); b) first three variables (shaded blue) are properties of populations, and the green shaded variables are properties of the environment; c) stronger selection around optimum reduces survival but promotes phenotypic change.

**Table S6.** General linear model (random effects) to predict population survival per generation. Combined *R^2^* = 0.66.

| Parameter^a^ | Regression coefficient | t-ratio |
| --- | --- | --- |
| Intercept | 441.2 | 37.0 |
| Heritability, *h^2^* | 49.3 | 16.7 |
| $\boldsymbol{\sigma}_{\boldsymbol{m}}^{\mathbf{2}}$ | 85.0 | 50.1 |
| Equilibrium $\boldsymbol{\sigma}_{\boldsymbol{g}}^{\mathbf{2}}$ | -142.4 | -81.5 |
| Strength of selection around optimum (-$\boldsymbol{\omega}$) | -216.3 | -204.5 |
| Environmental change per generation | -243.9 | -408.7 |

Notes: a) predictor variables are logarithmically transformed to improve the linear fit. The regression coefficients measure change in population survival for a unit change in log(predictor), controlling for all other variables. The strength of their contribution to the overall *R^2^* is evidenced by the *t*-test.

**Table S7.** General linear model (random effects) to predict standardised phenotypic change per generation. Combined R^2^ = 0.81.

| Parameter^a^ | Regression coefficient | t-ratio |
| --- | --- | --- |
| Intercept | 0.62 | 30.1 |
| Heritability, *h^2^* | 0.11 | 16.7 |
| $\boldsymbol{\sigma}_{\boldsymbol{m}}^{\mathbf{2}}$ | 0.07 | 25.2 |
| Equilibrium $\boldsymbol{\sigma}_{\boldsymbol{g}}^{\mathbf{2}}$ | 0.54 | 179.5 |
| Strength of selection around optimum (-$\boldsymbol{\omega}$) | 0.79 | 414.3 |
| Environmental change per generation | 0.45 | 330.1 |

Notes: a) all variables are log-transformed. The regression coefficients measure change in log(standardised rate of phenotypic change) for a unit change in log(predictor), controlling for all other variables. The strength of their contributions to the overall *R^2^* is evidenced by the *t*-test. The mutational variance influences the rate of phenotypic change via its effect on the equilibrium genetic variance with which it correlates (r = 0.87).

**Table S8.** General linear model (random effects) to predict the number of generations to extinction. Combined R^2^ = 0.75.

| Parameter^a^ | Regression coefficient | t-ratio |
| --- | --- | --- |
| Intercept | 5.65 | 121.1 |
| Heritability, *h^2^* | 0.08 | 19.4 |
| $\boldsymbol{\sigma}_{\boldsymbol{m}}^{\mathbf{2}}$ | 0.44 | 69.1 |
| Equilibrium $\boldsymbol{\sigma}_{\boldsymbol{g}}^{\mathbf{2}}$ | -0.73 | -110.2 |
| Strength of selection around optimum (-$\boldsymbol{\omega}$) | -0.55 | -129.0 |
| Environmental change per generation | -1.69 | -557.7 |

Notes: a) all variables are log-transformed. The regression coefficients measure change in log(standardised rate of phenotypic change) for a unit change in log(predictor), controlling for all other variables. The strength of their contributions to the overall *R^2^* is evidenced by the *t*-test.

The trajectory towards population extinction

Merely observing a population changing in step with time even over a period of decades isn’t sufficient to conclude that it is keeping up with the environmental optimum. To an outside observer without knowledge of the environmental optimum, the population in Figure S2 might give the impression of successful adaptation. But the selection resulting from the lag between the phenotypic mean and the environmental optimum (Figure S2a) causes the effective population size to decline (Figure S2b). A smaller effective population size increases the effects of genetic drift eventually bringing about wide swings in the phenotypic and genetic variances (Figure S2c) and weakening the response to selection.


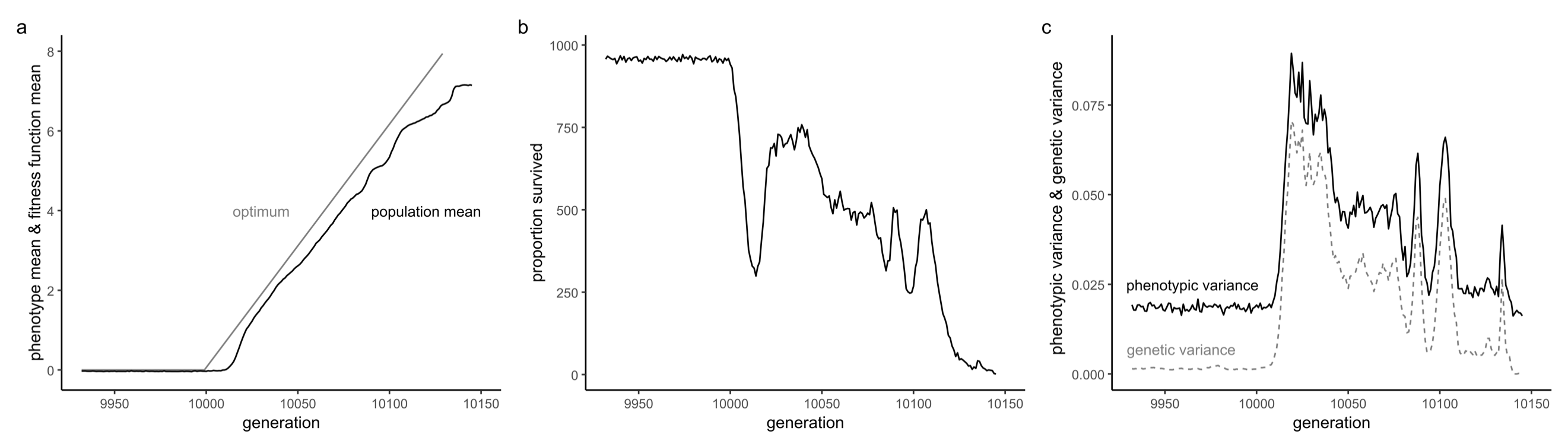


**Figure S2. The trajectory towards extinction in a single population.** The population experiences stabilising selection until generation 10,000 after which the environmental optimum is changed directionally. As theory predicts ^1^, a simulated population’s phenotype initially changes in parallel to a directionally increasing environmental optimum but with some lag, and then slows (panel a). The slowing coincides with high levels of mortality (panel b) which causes the effective population size to be low. At this point, the genetic variance, having initially risen in response to directional selection, has dropped and begins to fluctuate wildly owing to the effects of genetic drift (panel c). This increases lag, even fewer of the population survive selection (panels a,b), and the population eventually goes extinct as the genetic variance is depleted (panel c).

As the genetic variance becomes depleted, the population’s rate of adaptive change slows, increasing lag. At this point, the rate of decline in the number of generations a population survives actually accelerates as it lags further and further behind the optimum (Figure 1d caption, main text), indicating that lag progressively diminishes a species’ capacity to adapt. Even so, the phenotypic mean continues to change approximately linearly with the changing environment, still giving the impression of successful adaptation. But the population is actually falling progressively further behind the optimum leading to such a high rate of mortality that all members of the population eventually die in a single generation – and this last phase can occur precipitously.

Recovery from selection-induced mortality

Our simulations – like many artificial selection experiments (e.g., ^8-10^) – return populations to their ‘carrying capacity’ (fixed population size) each generation, but a question arises as to whether natural populations can recover from the levels of mortality that sustained long-term adaptation to a changing environment requires.

Estimates of the per capita population growth rate or $pgr=\frac{1}{n}\times\frac{dn}{dt}$, where *n* is population size and *t* is time, give the rate of growth starting from a very low population size and are available for a range of species^11,12^. The interest here is that population growth rate can be converted to an estimate of the proportion of the population that can be removed without causing the population to go extinct^13^: $p_{r}=1-\frac{1}{e^{pgr}}$ .

We calculated the average *p_r_* value for *n* = 719 species from 1849 time-series comprising *n* = 35,793 time-periods. Species are drawn from 16 Classes, including crustaceans, mammals, birds, bony fish, reptiles, insects and angiosperms, plus the phytoplankton^12^. On average $p_{r}=0.39\pm0.148$, indicating that species can suffer up to 39% ± 14.8% annual mortality (owing to all sources) and still rebound to carrying capacity (Figure S3a). For thirteen of the seventeen Classes plus the phytoplankton, 76% can accommodate up to 50% mortality (Figure S2b). One-hundred-fifty-six or 21.7% of the 719 species have maximum mortality scores that exceed 50%, just 59 or 8% can exceed 60% mortality and still recover, and only six or 0.8% exceed the 80% figure often used in artificial-selection experiments: a cyanobacterium, moth, vole, shrew, passerine bird, and lemming (none of these data was used to calibrate the simulations).


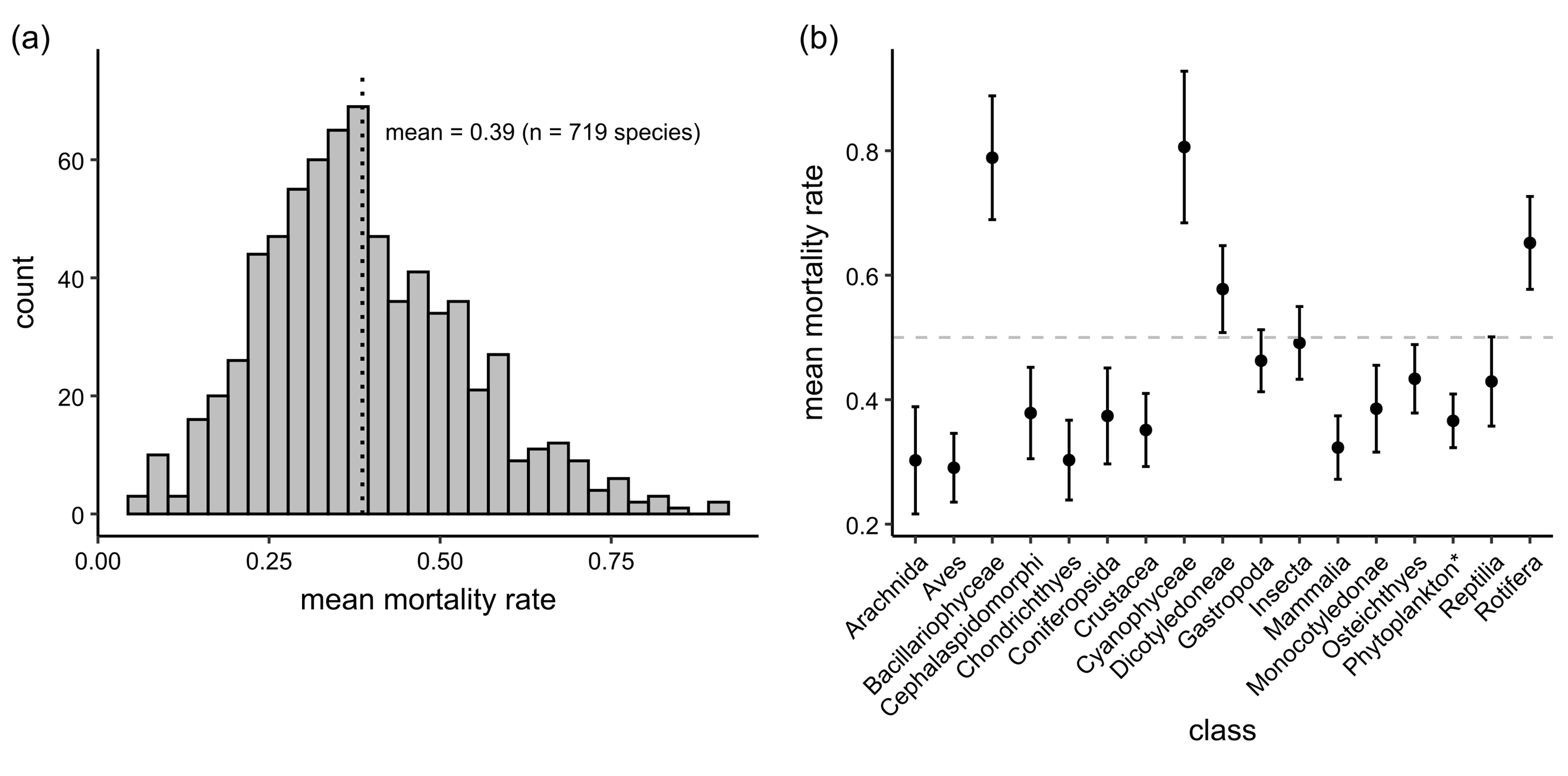


**Figure S3. Demographic thresholds for population recovery.** a) data for panels a,b from reference^12^: the maximum mortality (proportion) that a population can sustain and still recover to carrying capacity, n = 719 species from 16 Classes plus the phytoplankton* (* = not monophyletic); b) mean maximum mortality ± 1 standard deviation for 16 Classes plus the phytoplankton. Population Growth Rate (PGR) data were taken from Sibly et al.^12^ and are available upon request from the corresponding author of that paper.

These results suggest that if the high rates of long-term change that have been witnessed in some artificial-selection studies are unusual, they might also be unsustainable for most species in the wild, save for a few with fast life histories: the maize and *Drosophila* bristle number artificial-selection studies (main text) that achieved high rates of sustained change^8,10^ selected the top 20% of individuals (80% selective death) on the trait each generation, and the flying speed study selected roughly the upper 4.5%^9^, before artificially returning them to their pre-selection population sizes.

**Supplementary References**

1 Bürger, R. & Lynch, M. Evolution and extinction in a changing environment: a quantitative‐genetic analysis. *Evolution* **49**, 151-163 (1995).

2 Bürger, R., Wagner, G. P. & Stettinger, F. How much heritable variation can be maintained in finite populations by mutation–selection balance? *Evolution* **43**, 1748-1766 (1989).

3 Falconer, D. S. *Introduction to Quantitative Genetics*. 2nd edn (Longman, New York, 1981).

4 Huey, R. B. & Kingsolver, J. G. Evolution of resistance to high temperature in ectotherms. *The American Naturalist* **142**, S21-S46 (1993).

5 Endler, J. A. *Natural selection in the wild*. (Princeton University Press, 1986).

6 Kingsolver, J. G. & Pfennig, D. W. Patterns and power of phenotypic selection in nature. *Bioscience* **57**, 561-572 (2007).

7 Kingsolver, J. G. & Diamond, S. E. Phenotypic selection in natural populations: what limits directional selection? *The American Naturalist* **177**, 346-357 (2011).

8 Dudley, J. From means to QTL: The Illinois long‐term selection experiment as a case study in quantitative genetics. *Crop Science* **47**, S-20-S-31 (2007).

9 Weber, K. Large genetic change at small fitness cost in large populations of drosophila melanogaster selected for wind tunnel flight: rethinking fitness surfaces. *Genetics* **144**, 205-213 (1996).

10 Yoo, B. Long-term selection for a quantitative character in large replicate populations of Drosophila melanogaster: II. Lethals and visible mutants with large effects. *Genetics Research* **35**, 19-31 (1980).

11 Hone, J., Duncan, R. P. & Forsyth, D. M. Estimates of maximum annual population growth rates (rm) of mammals and their application in wildlife management. *Journal of Applied Ecology* **47**, 507-514 (2010).

12 Sibly, R. M., Barker, D., Denham, M. C., Hone, J. & Pagel, M. On the regulation of populations of mammals, birds, fish, and insects. *Science* **309**, 607-610 (2005).

13 Caughley, G. *Analysis of vertebrate populations*. (Wiley, New York, 1977).
